# A comparative study of coping skills and body image: Mastectomized vs. lumpectomized patients with breast carcinoma

**DOI:** 10.4103/0019-5545.43051

**Published:** 2005

**Authors:** Fiona Mahapatro, Shubhangi R. Parkar

**Affiliations:** * Associate Professor, Department of Psychiatry, Padmashree Dr D.Y. Patil Medical College and Rajawadi Hospital, Mumbai; ** Professor and Head, Department of Psychiatry, Seth G.S. Medical College & K.E.M. Hospital Mumbai

**Keywords:** Coping skills, body image, breast carcinoma, mastectomy, lumpectomy

## Abstract

**Background:**

The diagnosis of breast cancer encompasses not only physical, but also social and psychological implications because of the importance of the breast in a woman's body image, sexuality and motherhood. Women may experience a range of concerns and fears including physical appearance and disfigurement, the uncertainty about recurrence and the fear of death. There are no Indian studies on this subject.

**Aim:**

This study explores the various concerns of mastectomized and lumpectomized (breast conserved) patients, determines the coping mechanisms employed and the resolution of concerns. The levels of anxiety and depression in both groups were also studied.

**Methods:**

Seventy-five patients with breast carcinoma (50 mastectomized and 25 lumpectomized) were evaluated. The concern and coping checklist of Devlen was used. The severity of anxiety and depression was measured using the Hospital Anxiety and Depression Scale (HADS).

**Results:**

Body image or disfigurement was a concern only in the mastectomized group. Concerns were equally resolved between the two groups except for sexual role and performance, wherein the concern was resolved to a lesser extent in the mastectomized group. Coping strategies employed were effective in the resolution of concerns except for sexual role and performance, and recurrence or relapse. No statistically significant difference was found in the depression and anxiety levels of the two groups.

**Conclusion:**

Concern regarding sexual role and performance was resolved to a lesser extent in the mastectomized group. Specific psychological intervention is necessary to enhance coping strategies with regard to concerns of body image, and sexual role and performance.

## INTRODUCTION

Today, breast cancer is one of the most topical subjects in oncology, being the most common malignancy and a leading cause of death due to cancer in women. It is a well-established fact that a diagnosis of breast cancer has not only physical, but also social and psychological implications because of the importance of the breast in a woman's body image, sexuality and motherhood.^1^

Various psychosocial concerns have been reported in breast cancer. These concerns can lead to psychosocial distress, principally in the form of anxiety and depression. If such concerns can be elicited and resolved, psychiatric morbidity could be avoided.^2^

The manner in which different women respond to the diagnosis of breast cancer varies enormously. These variations may be due to individual coping strategies, personality factors, the level of social support available to them and, to a large extent, the consultation skills of her medical carers, especially the surgeon who breaks the bad news.

Patients have to take potentially complex decisions about treatment options, which are not helped by the changing policies for radical versus conservative interventions, and the lack of firm evidence to direct the choice of treatment.

## REVIEW OF THE LITERATURE

### Epidemiology

In the UK, breast cancer affects 1 in 12 women, accounting for 19% of all new cases of cancer among women.^3^ In India, the age-adjusted incidence figures for breast cancer published by the National Cancer Registry for 1984 for three areas (Mumbai, Bangalore, Madras) are reported to be 23.07, 20.4, and 18.4 per 100,000 female population, respectively.^4^,^5^ In the UK, approximately 15,000 die of breast cancer every year (5% of all female deaths).^3^ In India also, nearly 15,000 women die of breast cancer each year.^4^

### Psychopathology

Being diagnosed with and treated for breast cancer is an emotionally distressing experience for most women. They may experience a range of concerns and fears, which include physical appearance and disfigurement, uncertainty regarding recurrence and fear of death.^6^–^8^ These concerns can lead to psychological distress, principally in the form of anxiety and depression, which may persist for months after medical treatment has ended.^9^,^10^

Conventional wisdom suggests that conserving the breast would mitigate the psychological difficulties associated with breast cancer. However, a few studies that have compared the psychological effects of the two procedures (mastectomy and lumpectomy) have produced mixed results. Some researchers found lumpectomy to be superior^11^ while others found no difference between the two treatment modalities.^12^–^14^

Harrison and Maguire^15^ observed that younger patients were more likely to report concerns relating to the illness itself, treatment, feeling different, feeling upset, the future, finances, relationship with the partner and others, and sexuality. In contrast, older patients appeared to cope better. The reason attributed was that while the threat to life would have reminded older patients also of unfulfilled ambitions, they may have already faced existential threats to themselves or others and developed appropriate coping mechanisms.^15^ Ganz *et al*.^16^ suggested that older patients would also have had more experience of illness and in dealing with medical situations. They were less likely to experience job-related problems but there was no effect of age on problems of body image, worry about cancer, or marital and sexual problems.

The psychological outcome of cancer surgery has been found to be affected by the patient's premorbid personality and coping style, presence of stressful life events, severity of preoperative psychological morbidity, social support available and, in breast cancer patients, extent of surgery.

### Coping

Coping has been suggested as an important determinant of adaptation to cancer, both in terms of psychological morbidity and survival time. Significant associations were demonstrated between coping style and survival rates,^17^ but this was not invariably so,^18^ and much of the research has been criticized on methodological grounds.

Tunks and Bellissimo in 1988 commented on the theoretical, semantic and scientific limitations of attempting to define coping.^19^ The following are the definitions of coping given by various researchers.

1. Adaptation to a demanding situation^20^
2. Defence mechanisms that are stable and generalized traits^21^
3. Cognitive and behavioural efforts to master, reduce or tolerate internal and external demands, and conflicts among them that tax or exceed the person's resources^22^
4. Action directed at the resolution or mitigation of a problematic situation^23^
5. The attempt to ward off, to reduce or to assimilate an existing or expected demand (or stressor) either by intrapsychic effort (cognition- or emotion-related) or by action (field-related)^24^

### Coping styles

Morris *et al*.^25^ and Greer and Watson^26^ identified different coping styles. These have been described in terms of the five dimensions of mental adjustment to cancer such as fighting spirit, helplessness/hopelessness, fatalism, anxious pre-occupation and avoidance.

The concept of coping as a situation-specific response tends to focus on particular life events as sources of stress.

Three styles of coping strategies were described by Endler and Parker.^27^ (i) *Task-oriented strategies* attempt to solve a problem, reconceptualize and minimize the effect of the problem; (ii) *emotion-oriented strategies* include emotional responses, self-protection and fantasizing reactions; and (iii) *avoidance-oriented strategies* avoid stress by seeking out other people (social diversion) or by engaging in substitute tasks (distraction).^27^

Thomas and Marks^28^ demonstrated that acceptance and positive reframing were the two foremost coping strategies and the next five most common coping strategies were active coping, social support, religion, self-distraction and planning.^28^

Ali and Khalil^29^ categorized coping strategies into four groups—faith, compliance with the medical regimen, seeking information and social support, and self-distraction. Coping strategies expressed a combination of affect and direct action strategies, and were a reflection of the degree of religious adherence adopted by patients in enhancing their ability to cope with cancer. The participants reported five stressors—hope for cure, treatment effectiveness, fear of the unknown, progression of the disease and pain.^29^

Singh and Pandey^30^ found that the coping dimensions vary with the nature of the problems faced. Albuquerque *et al*. found sex differences among college students in the type of coping behaviour noted.^31^

### Body image

Body image can be described as a mental construct or picture that develops from infancy as a result of sensory and motor development, and exploration of the world around the individual.^32^,^33^

Studies have noted that there is a correlation between altered body image, poor self-esteem and psychological morbidity (depression and anxiety states) in individuals with breast cancer.^34^–^37^

A retrospective study by Fallowfield *et al*.^38^ questioned the view that mutilating treatment is predominantly responsible for the measurable psychiatric morbidity. In their study, they found that the incidence of anxiety and/or depression was 33% in mastectomized patients and 38% in breast-conserved patients.^38^

### Aims and objectives

1. To explore the various concerns associated with mastecto-mized and lumpectomized patients
2. To compare coping mechanisms and body image disturbances in both the groups
3. To determine the concerns, coping mechanisms employed and extent of resolution of concerns
4. To study the level of anxiety and depression in mastectomized and lumpectomized patients.

## METHODS

### Subjects

The subjects selected for this study were patients with breast cancer, visiting an oncology outpatient clinic in a cancer institute. All these patients were randomly selected as participants for this study during their follow-up visit; the study was for a period of one year. The sample consisted of 75 breast carcinoma patients, 50 of them had undergone mastectomy while 25 of them were treated conservatively (lumpectomy) for the same. The following inclusion and exclusion criteria were used for selection.

#### Inclusion criteria

- Diagnosis of breast carcinoma
- Postoperative period of 6 months to 1 year
- Age group of 18–50 years
- Language compatibility
- Educated up to the secondary level

#### Exclusion criteria

- History of any psychiatric disorder, as per DSM-IV guidelines
- History of any major medical illness (e.g. epilepsy, stroke, etc.)
- Breast cancer metastasis
- Recurrence of breast cancer

The selected sample (both mastectomized and lumpectomized patients) was interviewed with the help of a structured proforma, while they were waiting for their outpatient appointment. They were assured of complete confidentiality and key relatives were interviewed for data confirmation wherever needed.

#### Concern and Coping Checklist by Devlen

The patients' concerns were explored using a checklist developed by Devlen^39^ and shown to be reliable in other studies.^40^ The checklist was originally developed with a sample of lymphoma patients but has been successfully used with other cancer groups.^41^,^42^

Each patient was questioned on all the items of this checklist to discover whether it was a concern or not and if yes, the severity (mild, moderate, severe). An enquiry into additional specific concerns if any, was made. After a few clinical interviews, the two additional concerns included in the checklist were (i) interference in social activity, and (ii) recurrence or relapse. Further, for each concern, patients were questioned about the coping mechanism utilized to handle the concern, out of the 32 various possible coping mechanisms described by Devlen.^39^ The efficacy of coping was then evaluated on the basis of their perceived ability to resolve the concern and was rated as ‘the degree of resolution’ (complete, partial or none).

#### Hospital Anxiety and Depression Scale

All subjects were also administered the Hospital Anxiety and Depression Scale (HADS) to measure the severity of anxiety and depression. The caseness values were determined, a cut-off score of 7 was used for the anxiety subscale, a cut-off score of 8 for the depression subscale and a cut-off score of 16 for the total score.^43^,^44^

The data thus collected were analysed using the SPSS/ PC+ (statistical package for social sciences) package.

## RESULTS

### Sample characteristics

In both the mastectomy and lumpectomy groups the sample consisted of middle-aged women on an average. The mean ages (SD) of the women in the mastectomy and lumpectomy groups were 42±7.14 and 42.74±6.23 years, respectively. All the subjects were found to have a higher than secondary level of education as it was an inclusion criterion to ensure appropriate understanding of the Concern and Coping Checklist administered.

The majority of the women in both the groups were married, housewives (the rest held clerical to legal jobs), from a nuclear family and from the middle socioeconomic class.

Table 1 lists the most frequent among the 15 concerns assessed in the study. The less frequent concerns were subjective physical health, treatment, not being able to do things, job, finances, relationship with partner, relationship with others, support from the family, and interference in social activity.

**Table 1:** Predominant concerns in the lumpectomized and mastectomized groups (out of all 15 concerns)

Concern	Lumpectomy (*n*=25)	Mastectomy (*n*=50)
Current illness	15 (60)	32 (64)
Feeling upset or distressed	9 (36)	20 (40)
The future	9 (36)	21 (42)
Body image or disfigurement	0	39 (78)
Recurrence or relapse	15 (60)	41 (82)

Values in parentheses are percentages.

**Fig. 1:**
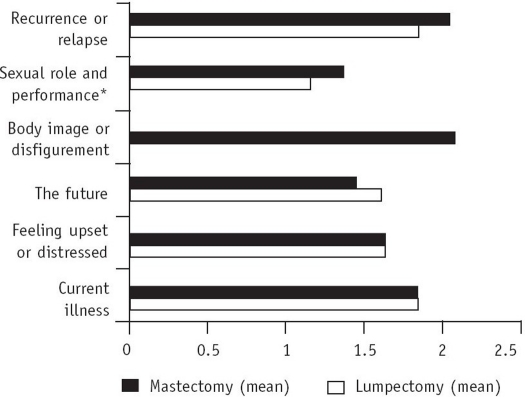
Statistical comparison between the lumpectomized and mastectomized groups of six important concerns using the unpaired *t* test

As seen in Figure 1, of the six important concerns, sexual role and performance concern showed a statistically significant difference (p<0.05) between the lumpectomized and mastectomized groups. The mean (SD) values were 1.12±0.44 and 1.38±0.56, respectively.

As shown in Table 2, in all the six concerns in both the groups, except for the concerns regarding body image or disfigurement and recurrence or relapse in the mastectomized group, there was a statistically significant increase, indicating that the coping strategies employed were effective in resolving these concerns.

**Table 2:** Statistical comparison between concern and resolution of the six frequent concerns of the lumpectomized and mastectomized groups by paired *t* test

Concern and resolution	Lumpectomized Mean (SD) *t*↑↓p value	Mastectomized Mean (SD) *t*↑↓p value
Current illness	1.8±0.76	1.80±0.70
	*t*=2.09↑↓*	*t*=0.04↑↓**
Resolution	2.8±1.84	2.7±1.73
Feeling upset or distressed	1.6±1.061	1.68±0.93
	*t*=3.91↑↓***	*t*=5.87↑↓***
Resolution	3.7±1.71	3.72±1.60
The future	1.6±0.91	1.48±0.61
	*t*=4.30↑↓***	*t*=6.62↑↓***
Resolution	3.7±1.71	3.60±1.69
Body image or disfigurement	All the values are 0	2.08±0.72
		*t*=0.75↑↓NS
Resolution		2.30±1.51
Sexual role and performance	1.12±0.44	1.38±0.56
	*t*=14.51↑↓***	*t*=7.73↑↓***
Resolution	4.76±0.83	3.82±1.68
Recurrence or relapse	1.80±0.70	2.08±0.69
	*t*=2.98↑↓**	*t*=0.22↑↓NS
Resolution	2.78±1.73	2.02±1.47

* p<0.05;

** p<0.01;

*** p<0.001; NS: not significant

**Fig. 2:**
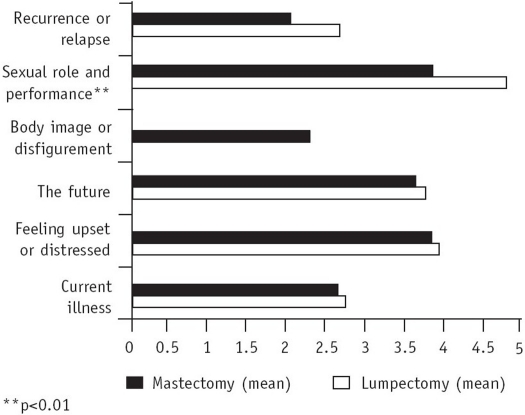
Comparison of the resolution of concerns between the lumpectomized and mastectomized groups using unpaired *t* test

Figure 2 shows that there was no statistically significant difference between the resolution of concerns in the two groups except for sexual role and performance, where resolution in the mastectomized group was to a lesser extent than the lumpectomized group (p<0.01; statistically significant).The mean (SD) was 3.82±1.68 and 4.76±0.83, respectively. In this particular resolution, the mean score for the lumpectomized group was significantly higher than for the mastectomized group, indicating that resolution was less often seen in the mastectomized group than the lumpectomized group. As all the values are zero for concern regarding body image in the lumpectomized group, no statistical test can be applied to detect differences between the two groups.

**Fig. 3:**
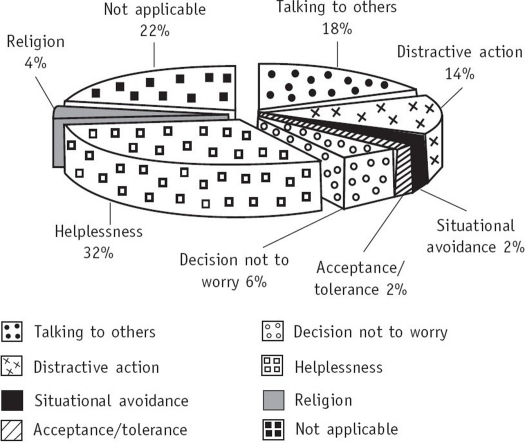
Coping strategies (most frequently) used for concern regarding body image or disfigurement in the mastectomized group

From Fig. 3 it can be seen that helplessness, talking to others, family and friends, and distractive action were the most frequently used coping strategies for the concern regarding body image in the mastectomized group. Other coping mechanisms were observed for the other concerns. It would be a Herculian task to mention all those here. In the mastectomized group, the coping strategy most frequently used for the concern regarding sexual role and performance was temporary acceptance (22%) and for that regarding recurrence or relapse, it was talking to others, mainly professionals (28%).

Table 3 shows no statistically significant difference between the two groups for hospital anxiety and depression scores.

**Table 3:** Statistical comparison of the Hospital Anxiety and Depression scores between the lumpectomized and mastectomized groups using unpaired *t* test

	Lumpectomy Mean (SD)	Mastectomy Mean (SD)	p value
Total anxiety score	0.92±1.68	1.66±1.74	0.082 (NS)
Total depression score	2.96±4.29	4.50±4.75	0.164 (NS)

NS: not significant

## DISCUSSION

This study emphasizes that patients with breast carcinoma have numerous concerns (Table 1). These concerns were in accordance with the findings of many research workers in patients with various types of cancer.^40^,^42^ The concern about sexual role and performance (Fig. 1) was more obvious in the mastectomized group, and reflects the women's perception regarding feelings of sexual attractiveness and desire.^14^ This is important, as 25% of women with breast cancer reported sexual problems.^45^

In the lumpectomized group there was no concern regarding body image or disfigurement. This could be attributed to the fact that the perception of feminity was preserved with conservation of the breast. In many other studies, the findings were mixed in nature, some showing no difference between the two treatment modalities and some supporting the hypothesis that breast-conserved patients have less psychological morbidity.^12^–^14^ Disturbance of body image and sexuality have also been well documented in many other studies in women with breast cancer.^9^,^12^,^25^

Interestingly, in this study, the coping strategies employed were helpful in resolving the concerns in each of the groups (Table 2). However, helplessness (32%), which was the most frequently used coping strategy for concern regarding body image in the mastectomized group was ineffective in resolving the concern. Helplessness is reported to be negatively correlated with well-being and has been documented as an ineffective coping strategy in other studies.^42^,^46^

Another strategy used was talking to others, family and friends (18%). As seen in various other studies, women approach their partners, close relatives and friends as their informal helpers.^47^

In the mastectomized group, the concern regarding recurrence or relapse concern, was not effectively resolved; the coping strategy most frequently used was talking to others, mainly professionals (28%). This reflects the persistence of worry, anxious preoccupation with the disease and efficacy of treatment like surgery. Uncertainty of recurrence of cancer was also found to be of greatest concern by other researchers.^47^ It should also be noted that going through mutilating surgery is itself a traumatizing experience that leads to severe psychological disturbance, hence adaptive processes seem to be incomplete and inadequate, further impairing the coping mechanisms.

Among the two groups, the concerns were equally resolved except for a statistically significant difference seen in the concern regarding sexual role and performance (Fig. 2) in which the mastectomized group resolved to a lesser extent than the lumpectomized group. The coping strategy most frequently used for this concern was temporary acceptance (22%). How these women cope up with this concern requires to be sensitively and sensibly explored in terms of their orgasmic capacity, sexuality practices including caressing, and the attitude of the partner. It is also essential for patients to know that loss of the breast need not necessarily contribute to sexual dysfunction, but the premature menopause brought on by systemic therapies (chemotherapy and hormonal therapy) may lead to sexual morbidity.^45^ This necessitates enhancement of the coping skills through pre- and post-operative counselling.

In this study, no statistical difference was found between the two groups for HADS scores. A similar finding was reported in other studies, wherein both the groups experienced similar levels of psychiatric morbidity.^48^,^49^ Coping styles are of particular importance, both as significant predictors of disease outcome and prognosis.^1^,^17^,^42^

Thus, it is the fear of cancer that gives rise to the symptoms of depression and anxiety, irrespective of the treatment modality. This highlights the role of psychosocial interventions in helping such women deal with concerns and the coping styles they employ.

Identification of the most common concerns faced by women with breast carcinoma is essential to formulate better intervention measures to deal with these concerns. Specific psychological intervention, especially in enhancing coping strategies employed by them is needed in the areas of body image and sexual role and performance. Preoperative and postoperative counselling should include these concerns. It should also examine the depression and anxiety related to the fear of cancer and, wherever necessary, the use of psychotropic medications. This will not only contribute in reducing the mental health problems of these women but also improve their quality of life and subjective feeling of well-being. A specific focus on cultural context will be an important area for future research.
